# Heritability for stroke: Essential for taking family history

**DOI:** 10.22088/cjim.11.3.237

**Published:** 2020-05

**Authors:** Masoumeh Pourasgari, Ashraf Mohamadkhani

**Affiliations:** 1 Cell and Molecular Biology Department, Faculty of Biological Sciences, Kharazmi University, Tehran, Iran; 2 Liver and Pancreatobiliary Diseases Research Center, Digestive Disease Research Institute, Shariati Hospital, Tehran University of Medical Sciences, Tehran, Iran

**Keywords:** Family history; Stroke, Pedigree, Stroke genes

## Abstract

**Background:**

There are many well-established factors that influence the risk of stroke including blood pressure, diabetes, low socioeconomic status and smoking, however, the shared genetic resource in members of a family effect on stroke predisposition. Genome-wide association studies (GWAS) have demonstrated evidence of a shared genetic source in stroke risk. This review considered the influence of family history as one of the main risk factors in stroke according to the literature.

**Methods:**

Literature review was obtained by searching for the key words "stroke", "family history" and "stroke gene" in PubMed. An overview has been made on the topics: relevance of stroke family history, family history assessment tools and specific candidate genes for stroke.

**Results:**

Family history of stroke is an important risk factor for the development of cerebrovascular diseases in addition to stroke subtypes in relatives who have reached the questionnaire and pedigree. While variation in a small number of loci showed Mendelian inheritance of stroke phenotypes, the genetic variations in several stroke risk loci are shared with multiple related vascular traits.

**Conclusion:**

This study highlighted the importance of family history in stroke phenotypes and current related genetics information. Increasing awareness of the importance of family history in stroke has the advantage of preventing exposure to stroke with health care.

Stroke is an acute neurological event that has been characterized by loss of motor, sensory and cognitive function (1, 2). Strokes occur when blood flow to the brain breaks down. There are three main types of stroke including ischemic, hemorrhagic and transient ischemic attack. Nearly 20% of strokes are hemorrhagic, causing bleeding in the brain (3). Some of the major treatable risk factors for stroke are: hypertension, cigarette smoking, signs of heart disease or history of stroke, diabetes, imbalance of cholesterol, physical inactivity and obesity (4). A risk factor is a situation or behavior that usually occurs in those who develop a higher risk of having a disease than those who do not (5). By increasing the number of risk factors, the risk of stroke increases. Therefore, it is essential to develop well-organized methods to prevent or reduce the occurrence of stroke (6). Recent progress in the treatment of stroke, in addition to the control of certain risk factors such as blood pressure, salt intake, weight and smoking can reduce the tendency of mortality (7). However, some factors for stroke cannot be changed by medical treatment or lifestyle changes. Although the greatest attention has been focused on environmental effects and lifestyle, since they can be adapted and controlled, it has been documented that the stroke also has a hereditary base that can interact with environmental factors (8). The family history of a premature stroke is an independent risk factor for stroke inheritance and with greater force in subjects between 25 and 49 years (9, 10).

Inheritance is an estimated measure of the genetic contribution to the total phenotypic trait (11, 12). Therefore it has been shown that a positive family history of stroke may be a signal for the onset of stroke at a younger age; however, large variations in age at onset indicate that environmental elements are also important in determining whether a person can progress with stroke (1, 13-15). To advance our understanding of the effects of genetics and the aggregation of family history as a risk factor in stroke, we have summarized the recent findings in these characters.

**Acquisition of evidence: **The Medline literature was searched by the key words "stroke", "family history", "pedigree" and "stroke gene" until August 2018 to study the genetic risk factors for stroke. A narrative overview was performed on the relevance of family history in stroke and family history assessment tool. Further revisions have been recognized by examining the appropriate bibliography to which reference is made in the original documents.

**Family history report to caress: **Several prospective cohort and case-control studies have examined the coherent relationship in the family history of stroke (9, 10, 13, 14). The results of these studies are sometimes inconclusive and may be the consequence of alterations in the design of the study, approaches to the recognition of stroke in family members, insignificant sample size or selection of the study population (14, 16). Population-based prospective cohort studies have shown a positive family history as a risk factor for stroke development. Family history was more strongly associated with young age in both hemorrhagic and ischemic stroke. However, as people grow older, there are fewer effects of a family history and genetic factors at the stroke risk (14, 17, 18). Nonetheless, an increased risk of stroke has been reported in those children who have a family history of stroke before age 65 (9). It has been shown that the presence of a family history of stroke in at least one parent doubled the risk of stroke among men and increased the risk among women (18-20). In this way, a stroke history in a sibling is also an increased risk of stroke in a proband (17). Female patients are more expected to have a stroke of first degree in particular with maternal history than in male patients (20). However, there is no variation of the paternal with the maternal history of stroke in male probands (20). Genetic factors, related to age under environmental impacts, explain sex differences due to the risk of stroke. An increasing risk of stroke is also observed in monozygotic twins compared to dizygotic twins or siblings (13). However, rare models of Mendelian stroke are also reported in monogenic disorders of sickle cell anemia and Fabry’s disease (5, 21). 

**Tools for collecting the family history: **Clinical disorders in adults indicating family inheritance may progress or delay the onset of the disease in adjustments to environmental factors (22, 23). Genetic variants allow the accumulation of phenotypes of familial diseases in individuals of a given population. The achievement of family history is a record of information on the health of the person participating as the main point of a family (proband) and its close relatives for genetic study and the cause of death in three generations (24). Family history analysis provides risk assessment in primary care practices and helps screening, diagnosis and early therapy, which can prevent or improve disease outcomes in individuals and their close relatives and suggests an opportunity for promotion of health (23, 24). Several methods have been recognized for obtaining family clinical histories, each with its specific advantages and disadvantages (25). The family history questionnaire that provides more accurate information is the usual approach in general practice. The appropriate response on the questionnaire is verified by the health care provider for further information including the relationship of the family members with the patient, the accurate identification, age of onset and severity of the disease. Another tool for evaluating the family history used by the genetic counselor is the pedigree. A family tree preferably shows at least three generations with the use of standardized symbols. This tree obviously identifies affected individuals with a specific diagnosis. A family tree indicated the age of the individuals, the age and causes of death in the deceased and any appropriate clinical history or illness (23). The results of any genetic testing of family members, if it has been achieved, should be indicated on the pedigree. The pedigree analysis of populations of unselected patients regularly reveals numerous genetic risk factors that were not previously recognized (21, 26, 27). The presence of a younger first-degree relative affected by family history could increase the risk of illness two to five times in the general population, however, a strong family history of a dominant trait may bring the risk of a disease to 50% or others (28).

**Genetic variations that influence stroke heritability: **Stroke is a polygenic disease that is influenced by multiform mechanisms of the interaction of genetic and non-genetic factors (29). Genome-wide research has exposed stroke-related genes, with the function of development and regeneration of the peripheral nervous system, in the inheritance of intracerebral hemorrhage and ischemic stroke (30). However, due to the complexity of gene-gene and gene-environment interactions, they are not applied in clinical practice. Most of the family genetic studies have been conducted in European descendants, so the distribution and heritability of stroke may be different among the ethnic groups (31). A meta-analysis of multi-ancestry genome-wide association on approximately 8 million single nucleotide polymorphisms (SNPs), revealed SNPs with a minor allele frequency (MAF) ranged from 0.6% to 1.8% involved in stroke, although some showed a specific association for the particular population (32). The majority of stroke loci recognized in this meta-analysis showed a common genetic association with other vascular traits. These loci have potent association with cardiac mechanisms further than those of cardioembolism that is not previously implicated in stroke pathophysiology (32).

Mutations in three loci of HTRA1, COL4A1 and COL4A2 genes influence Mendelian inheritance for stroke phenotypes (33-35). However, common variants such as the A222V polymorphisms in Methylenetetrahydrofolate reductase, E298D of nitric oxide synthase (NOS3), R506Q mutation of the factor V Leiden and G20210A nucleotide substitution of prothrombin contribute to the risk of stroke. The polymorphism in the intron 16 of the angiotensin-converting enzyme (ACE) gene is an independent risk factor for spontaneous intracerebral hemorrhagic stroke (36). The genetic markers rs11833579 and rs12425791 on the 12p13 chromosome near or inside the NINJ2 gene are hereditary causes of family aggregation of stroke in the linkage analysis (37). Furthermore, the polymorphisms and haplotypes of TGF-β1 are significantly associated with the risk of ischemic stroke in the population of northern India (38). The loci rs2280887 and rs4376531 belonging to the ARHGEF10 protein coding gene are associated to atherothrombosis and ischemic stroke (39). The 3 prime UTR variant rs2507800 of ANGPT1 gene is involved in the stroke by interfering to the binding to miR211 (40). 

As a result, the risk of stroke within these loci estimates the hereditary risk and the potential of stroke genetics for the discovery of new drugs. Significant locations in the candidate genes for stroke risk that are mostly reported in the previous study (32-37) are listed in table 1, which shows the heritability of genetic variation as a risk factor for stroke syndrome.

In conclusion positive family history is the main risk factor for stroke at a young age. There are some conventional methods that deal with family medical histories, including the questionnaire and pedigree, which must be customized to prevent or reduce the risk of hereditary stroke. Although the hereditary medical condition is typically caused by monogenic disorders, genome studies in a series of prospective cohort and case-control studies have identified several genetic loci involved in stroke that make the role of family history questionable. Stroke risk loci shared genetic variations with multiple vascular diseases that significantly deepened in pharmacological targets for antithrombotic therapy. Despite numerous studies, investigations on inheritance of stroke require more genetic research to support the suggested role of the genetic component in stroke etiology in individuals with a positive family history.

**Table 1:** Effective stroke loci in candidate genes for stroke risk with function information

SNP	Chromosomal region	Nearby Gene	Function of encoding protein
**rs4932370**	15q26	FURIN-FES	FURIN is a membrane type-1 protease that is expressed in different tissues, including neuroendocrine, liver, intestine and brain.The proto-oncogene c-Fes / Fps has tyrosine-specific protein kinase activity that is vital in cell transformation.
**rs2229383**	19p13	ILF3-SLC44A2	Interleukin Enhancer Binding Factor 3 a double stranded RNA (dsRNA) binding protein regulates gene expression and stabilizes mRNAs.Solute Carrier Family 44 Member 2 is linked to the transport of glucose and other sugars, bile salts and organic acids, metal ions and amino compounds and the innate immune system.
**rs146390073**	1q43	RGS7	The Regulator of G-protein signaling 7 acts as a universal Gi /o-coupled GPCR inhibitor in the brain.
**rs12932445**	16q22	ZFHX3	Zinc Finger Homeobox 3is a transcription factor with homeodomans and zinc-finger motifs and regulates myogenic and neuronal differentiation.
**rs13143308**	4q25	PITX2	Paired-like homeodomain transcription factor 2 regulates the activity of other genes and as part of a family of homeobox genes that act during early embryo development.
**rs4959130**	6p25	FOXF2	Forkhead Box F2 is expressed in lung and placenta, transcriptionally activating several specific lung genes.
**rs2107595**	7p21	HDAC9–TWIST1	Histone deacetylase 9 alters the chromosome structure and influences access to the transcription factor of DNA. Twist-related protein 1 is a basic helix-loop-helix (bHLH) transcription factor essential in embryonic development and regulates the transcription of genes involved in closing the cranial suture during skull development.
**rs635634**	9q34	ABO	The Histo-blood group ABO system transferase is an enzyme with glycosyltransferase activity.
**rs12124533**	1p13	TSPAN2	Tetraspanin-2 mediate signal transduction events that play a role in regulating cell development, activation, growth, and motility
**rs2005108**	11q22	MMP12	Matrix metalloproteinases (MMPs) are involved in the breakdown of extracellular matrix in embryonic development, reproduction, and tissue remodeling, as well as in disease processes, such as arthritis and metastasis.
**rs3184504**	12q24	SH2B3	SH2B adapter protein 3, also known as lymphocyte adapter protein (LNK), is ubiquitously expressed in many tissues and cell types act as a regulator in the signaling pathways related to hematopoiesis, inflammation and cell migration.
**rs12445022**	16q24	ZCCHC14	Zinc Finger CCHC-Type Containing 14 is related to nucleic acid binding and phosphatidylinositol binding. Zinc Finger CCHC-Type Containing 14 act as nucleic acid binding and phosphatidylinositol binding.
**rs1052053**	1q22	PMF1–SEMA4A	Polyamine Modulated Factor 1 has transcription coactivator activity of and leucine zipper domain binding Semaphorin 4A is involved in the guidance of axons, morphogenesis, carcinogenesis and immunomodulation.
**rs10820405**	9q31	LINC01492	Long Intergenic Non-Protein Coding RNA 1492 is an RNA Gene, and is affiliated with the non-coding RNA class.
**rs12476527**	2p23	KCNK3	Potassium channel subfamily K member 3
**rs7610618**	3q25	TM4SF4	Transmembrane 4 L Six family members 4, also known as the tetraspanin family, are cell surface proteins that mediate signal transduction events and regulat cell development, activation, growth and motility.
**rs34311906**	4q25	ANK2	Ankyrin 2 (Ankyrin-B) shape the cell's structural framework (the cytoskeleton) and link certain proteins that span the cell membrane to this framework.
**rs17612742**	4q31	EDNRA	Endothelin Receptor Type A plays a role in potent and lasting vasoconstriction. This receptor is associated with the guanine-nucleotide binding (G) proteins and this coupling activates a phosphatidylinositol-calcium second messenger system.
**rs6825454**	4q31	FGA	Fibrinogen Alpha Chain is a preproprotein proteolytically processed by thrombin to fibrin in clotting process.
**rs11957829**	5q23	LOC100505841	A Zinc Finger Protein 474-Like.
**rs880315**	1p36	CASZ1	Castor Zinc Finger 1, a transcription factor, may function as a tumor suppressor and controlling cell fate, a Putative survival-related protein.
**rs12037987**	1p13	WNT2B	Protein Wnt-2b (formerly Wnt13) plays a role in human development as well as in human carcinogenesis. This gene produces two alternative transcription variants.
**rs6891174**	5q35	NKX2-5	Homeobox protein Nkx-2.5 with tissue-specific gene expression regulation functions in heart formation and development.
**rs16896398**	6p21	SLC22A7–ZNF318	Solute carrier family 22 member 7 involved in the independent transport of sodium and excretion of organic anions, some of which are probably toxic. Zinc Finger Protein 318 is related to Androgen receptor signaling pathway.
**rs42039**	7q21	CDK6	Cyclin Dependent Kinase 6, a member of the CMGC family of serine/threonine protein kinases, is important for cell cycle G1 phase progression and G1/S transition.
**rs7859727**	9p21	chr9p21	Interferon Omega 1 as an interferon with antiviral activity.
**rs2295786**	10q24	SH3PXD2A	SH3 and PX Domains 2A protein associated with Malignant Peripheral Nerve Sheath Tumor
**rs7304841**	12p12	PDE3A	Phosphodiesterase 3A involved in the regulation of contractility of cardiac and vascular smooth muscle. This protein mediates platelet aggregation and also plays important roles in cardiovascular function
**rs35436**	12q24	TBX3	The T-box transcription factor TBX3, important in the regulation of developmental processes, is a member of a family of genes that share a common DNA-binding domain, the phylogenetically conserved T-box.
**rs9526212**	13q14	LRCH1	Leucine Rich Repeats And Calponin Homology Domain Containing 1 protein may be associated with osteoarthritis.
**rs11867415**	17p13	PRPF8	Pre-MRNA Processing Factor 8, is a component of both U2- and U12-dependent spliceosomes, and found to be essential for the catalytic step II in pre-mRNA splicing process.
**rs8103309**	19p13	SMARCA4-LDLR	SWI/SNF Related, Matrix Associated, Actin Dependent Regulator Of Chromatin, Subfamily A, Member 4 is required for transcriptional activation of genes. Moreover, it mediates the endocytosis of in cholesterol-rich LDL and therefore maintains the plasma level of LDL.
**rs11833579**	12p13	NINJ2	Ninjurin 2 (Nerve Injury-Induced Protein 2) a cell surface adhesion protein that is upregulated in Schwann cells surrounding the distal segment of injured nerve
**rs12425791**	12p13	NINJ2	-
**rs2280887**	8p23.3	ARHGEF10	Rho Guanine Nucleotide Exchange Factor (GEF) 10 regulate the activity of small Rho GTPases by stimulating the exchange of guanine diphosphate (GDP) for guanine triphosphate (GTP) and may play a role in neural morphogenesis
**rs4376531**	8p23.3	ARHGEF10	-
**rs2507800**	8q22.3-q23	ANGPT1	Angiopoietin 1, a secreted glycoprotein belonging to the family of angiopoietins with important roles in vascular development and angiogenesis.
